# Supplementary material for: Creatinine versus cystatin C for renal function-based mortality prediction in an elderly cohort: The Northern Manhattan Study
Source: PLoS One. 2020 Jan 15;15(1):e0226509. doi: 10.1371/journal.pone.0226509 (PMC6961921; doi:10.1371/journal.pone.0226509)
Supplement: Attachment — Submitted filename: Reviewers comments-PLOS ONE final.docx [file pone.0226509.s001.docx]

We appreciate the reviewer’s comments on our manuscript. We have included the comments by the reviewers below and have modified the manuscript as requested in the appropriate sections and included below are responses to the reviews.

Reviewer #1:

1. Although there was a slight but significant difference in a predicting power between the two measures, the reason is not clear. As the authors state in Discussion, the most important point must be whether eGFRcys is superior in accurately measuring GFR in their cohort. This point should be clarified at least in a small number of subjects in their cohort, using iohexol or inulin as an exogenous marker.

Response:

We agree with the reviewer that having a gold-standard measure of GFR would be ideal. Unfortunately the serum measures were collected at the time of initial enrollment in the Northern Manhattan Study between 1993 to 2001 such that we do not have the ability to measure GFR concomitantly. We currently do not have funding to measure GFR objectively in NOMAS but this is a planned future study if funded. We have acknowledged this is a limitation of our study.

2. Also, the dot plot between eGFRcr (mean, 74.7) and eGFRcys (mean, 51.7) should be presented to know the correlation between the two. As the CKD stage progresses, does the discrepancy become larger?

Response:

We agree and have included the following figure to outline the raw data with a dot and Bland&Altman plot. From our data it seems the discrepancy between eGFRCys and eGFRCr is higher at the higher ranges of GFR estimation than in the lower end. We have included this as the new figure 1 and added a comment in the results section.

Dot plot (left) and the Bland & Altman plot (right) (figure 1)

In results section: “The mean eGFRcr (74.68±18.8 ml/min/1.73m^2^) was higher than eGFRcys (51.72±17.2 ml/min/1.73m^2^); there was a greater difference in GFR estimations at the upper rather than lower ranges (figure 1).”

3. It is also important and should be analyzed whether the predicting power of eGFRcys was influenced by the severity of CKD, i.e., CKD stage 1-2 (eGFR > 60), stage 3, or stage 4-5 (eGFR < 30). Mortality incidence should have been more often in advanced CKD stages. This point should be clearly presented.

Response:

We agree with the reviewer than analyzing by CKD stages would have been ideal in our analyses. Unfortunately the proportion of participants with stages 4-5 in our cohort was small and chose to collapse stages 3-5 together. We were nonetheless concerned that severity of CKD would be important and performed our analyses using GFR in a continuous manner (per 10 ml/min/1.73m^2^) and noted there was a significant association with all-cause mortality.

4. In Figure 3, the ROC curve of eGFRcys seemed much better than eGFRcr among the subjects under 70. Why?

Response:

We thank the reviewer for highlighting this finding in our study. We were concerned that in the older participants in our study serum creatinine would be less predictive due to loss of muscle mass in the elderly, as well as less validated GFR estimation formulae for multi-ethnic populations such as ours.

The discussion has been modified as follows:

“The results, particularly in the participants older than age 70, related to eGFR_cys_ are consistent with findings from other studies suggesting that eGFR_cys_ may be a more accurate estimate of GFR than a serum creatinine-based formula, and extend those findings to an elderly multiethnic population where GFR_cr_ may be confounded by loss of muscle mass which would attenuate the association. The inability to accurately estimate GFR disproportionately affects blacks and Hispanic elderly patients creating significant challenges for prognostication for outcomes, decline of renal function, and management (particularly for medication dosing) of these individuals.”

5. It is not clear what are critical conditions where eGFRcys is superior to eGFRcr in mortality risk prediction. Males? Younger age? Caucasians? Subgroup analysis should be performed in order to specify the factors which have impact in favor of eGFRcys for risk prediction.

Response:

We performed analyses examining GFR estimates by sex, age, and race-ethnicity and noted that for predicting 5 year mortality risk, eGFR_cys_ was better than eGFR_cr_ among those age under 70 years old (p for difference=0.047, compared to age>70) or men (p for difference=0.049, compared to woman). No race-ethnicity differences were found. We have included this in the results section.

For predicting 10 year mortality risk, there were no statistically interactions.

We have included the following table for reference for the reviewer but not in the manuscript since the results were outlined in text.

|  | 5 year mortality | | |  | 10 year mortality | | |
| --- | --- | --- | --- | --- | --- | --- | --- |
|  | NRI (%) | 95% CI of NRI | p for difference |  | NRI (%) | 95% CI of NRI | p for difference |
| age<70 | 22.3 | (10.6, 34.0) | 0.047 |  | 4.6 | (-2.5,11.8) | 0.654 |
| age>=70 | 9.3 | ( 4.2, 14.4) |  |  | 2.9 | ( 0.3, 5.4) |  |
|  |  |  |  |  |  |  |  |
| Women | 7.4 | (1.4, 13.4) | 0.049 |  | 4.4 | ( 1.2 ,7.6) | 0.965 |
| Men | 16.9 | (9.6, 24.3) |  |  | 4.5 | (-0.2, 9.2) |  |
|  |  |  |  |  |  |  |  |
| White | 8.8 | (0.7, 16.9) | 0.977 |  | 0.8 | (-3.5, 5.2) | 0.187 |
| Black | 12.1 | (4.1, 20.0) |  |  | 6.5 | ( 1.9,11.2) |  |
| Hispanic | 14.9 | (6.6, 23.2) |  |  | 4.9 | ( 0.2, 9.7) |  |

We have included the AUC’s in our figures to describe overall model fit in these groups. In conclusion serum cystatin-C based GFR estimated performed better in those under age 70 and in men. Overall however the AUC was low for both cystatin-C and creatinine based GFR estimations emphasizing the need for further better data in diverse populations such as the elderly and women. We have now included this as an additional comment in the results and discussion.

“Interestingly in our cohort the predictive ability of eGFR (regardless of serum measure) appeared higher in the younger participants and men who were most likely to be included in prior cohort that derived GFR estimation formulae. These results highlight the importance of improved accuracy in measurement of GFR in diverse populations will help better understand how CKD is associated with CVD mortality related disparities.”

Reviewer #2:

1. I believe that authors knew serum cystatin C concentration is influenced by many factors, hyper/hypo thyroid, HIV infection, and so on. I could not find in the manuscript about exclusion of these patient from cohort.

Response:

We thank the reviewer for this comment. We did not collect information on thyroid and HIV status is NOMAS, but these conditions were not exclusion criteria for the Northern Manhattan Study. Participants were excluded from a medical condition perspective only if they already had a stroke.

2. Main finding of this study may be “eGFRcys predicted all-cause mortality better than eGFRcre”. However, this fact is already reported indirectly as the authors cited in Ref 24, 33,34, and Astor BC et al 2011, Peralta CA et al 2011

Response:

We agree with the reviewer, however this topic has not been explored to the same degree in diverse, multi-ethnic, and more predominantly older populations such as the Northern Manhattan Study.
