# Supplementary material for: Creatinine versus cystatin C for renal function-based mortality prediction in an elderly cohort: The Northern Manhattan Study
Source: PLoS One. 2020 Jan 15;15(1):e0226509. doi: 10.1371/journal.pone.0226509 (PMC6961921; doi:10.1371/journal.pone.0226509)
Supplement: Attachment — Submitted filename: Reviewers comments-PLOS ONE v2.docx [file pone.0226509.s002.docx]

We appreciate the reviewer’s comments on our manuscript. We have included the comments by the reviewers below and have modified the manuscript as requested in the appropriate sections and included below are responses to the reviews.

Reviewer:

1. As a reviewer pointed out, cystatin C is affected by multiple conditions. It is a limitation of this study that you can not exclude those cohort with thyroid dysfunction, HIV infection and others.  The authors should describe the limitation on this point.

and

At least authors should refer in manuscript about my previous comment 1. Because it must exist and affect on the results.

Response:

We agree with the reviewer and editor that the lack of this kind of medical comorbidity information is a limitation of our study and have included the following sentence in the limitation sections:

“Cystatin-C levels can be affected by several medical conditions including thyroid dysfunction^44^ and human immunodeficiency virus infection^45^ which unfortunately we did not collect in NOMAS.”

We have also added the very helpful references by the reviewer on other studies that have studied creatinine and cystatin as predictors (Astor BC et al 2011, Peralta CA et al 2011).
